# Gambling among adolescents with and without hearing loss

**DOI:** 10.1186/s40405-016-0015-y

**Published:** 2016-08-04

**Authors:** Susanna Geidne, Karin Fröding, Madelene Larsson

**Affiliations:** Faculty of Medicine and Health, School of Health Sciences, Örebro University, Örebro, Sweden

**Keywords:** Gambling, Prevalence, Hard-of-hearing, Youth, Problem gambling

## Abstract

**Objectives:**

This exploratory study investigates the prevalence of gambling, preferred types of gambling, and problem gambling in Swedish young people aged 15–18 years with and without hearing loss.

**Methods:**

A cross-sectional health survey was conducted in Örebro County, Sweden in 2014. A standardized questionnaire was distributed to 4888 students, and 4329 filled it. There were 318 (8 %) students with hearing loss. The response rate was 82 %. The 2-item Lie/Bet questionnaire (Johnson et al. in Psychol Rep 80:83–88, 1997) was used for measuring problem gambling.

**Results:**

More students with hearing loss had gambled during their lifetime (35 %) and in the past year (25 %) than their hearing counterparts (lifetime: 24 %; past-year: 19 %). More students with hearing loss compared to normal hearing students were identified as problem gamblers (7.7 % compared to 4.3 %).

**Conclusion:**

More research is needed on gambling among people with hearing loss as well as other disabilities.

## Background

Adolescence is a time when many lifestyle behaviors like substance use and gambling are established (Currie et al. 2012; Lake et al. 2006). It is also a period of increased risk-taking, as adolescents seek to become independent individuals (Hanson et al. 2014). Young people’s risk-taking behavior, like gambling is an important area of research (Blinn-Pike et al. 2010; Messerlian et al. 2005) but data on gambling and health needs of young deaf persons are lacking (Barnett et al. 2011). This study tried to fill this research gap.

### Prevalence of gambling among young people with and without hearing loss

It is stated that the proportion of problem gamblers is higher among adolescents than among adults (Blinn-Pike et al. 2010). The proportion of disordered gamblers is estimated to almost 8 % among college students, although there were quite large variations between studies (Blinn-Pike et al. 2007). There is consensus that boys are more likely to gamble across all target groups and countries. Recent studies from the Nordic countries also find diverse prevalence rates of gambling for young people, ranging from 20 to 79 %. In Sweden, between 20 and 33 % of boys aged 15–17 years (Svensson 2014) gambled in the past 12 months. In Iceland around 57 % of 13–18 year olds, both boys and girls (Olason et al. 2011) gambled. In Norway 79 % was noted (Hansen and Rossow 2008). In Finland 52 % of a sample of 12–15 year olds (Castrén et al. 2015) had gambled in the past 12 months. When it comes to problem gambling, the definitions differ leading to proportions between 0.2 and 21 %. Problem gambling among 16–17 year olds in Sweden was estimated to be around 5 % in 2009 (Abbott et al. 2014). Both the 0.2 % and the 21 % approximation come from Norwegian samples (Hansen and Rossow 2008; Hanss et al. 2015). Around 2 % of 13–18 year olds in Iceland were found to be problem gamblers (Olason et al. 2011).

Young people with learning disorders or special education needs showed a higher risk of developing problem gambling (Parker et al. 2013; Taylor et al. 2015). There have also been studies concluding that minority groups have higher rates of problem gambling (Breen and Gainsbury 2013). People with hearing loss can be considered a minority group, with some even having sign language as their first language (Barnett et al. 2011). Self-Categorization Theory also suggests that all social groups have their specific norms and that people tend to categorize themselves as similar to the members of one social group and that this also may affect an individual’s behavior (Hogg 2000; Hornsey 2008; Widén 2006). Only one study found connects between gambling and hearing loss and concludes that a higher proportion (35 % vs 18 %) of adolescents with a hearing disability in combination with other disabilities had gambled in the last 30 days than of those who are hard-of-hearing but have no other disability (Brunnberg et al. 2007).

Internationally, peer-related activities such as card-games and betting on sports are popular among young people (Volberg et al. 2010). Online gambling, which is easily accessible for children and adolescents (Parker et al. 2013), has an annual growth rate of almost 15 % (European Commission 2012). Among 16–24 year olds in Sweden, internet gambling increased from 11 to 18 % during 2008–2009 (Svensson and Romild 2011). The hearing loss group is at particular risk because online activities could be a more comfortable setting for this group (Blom et al. 2014). Young people often start gambling through social processes within significant social networks (Kristiansen et al. 2015).

### Health consequences of problem gambling

Problem gambling is a public-health problem (Algren et al. 2015; Griffiths 2004; Swedish Public Health Agency 2014), especially for adolescents and young adults (Messerlian et al. 2005; Volberg et al. 2010). Social and health costs of problem gambling are large both for societies and individuals (Griffiths 2004). In Sweden gambling is prohibited by law for persons less than 18 years, and in casinos the age limit is 20 years (Swedish Government 1994).

Health consequences among young gamblers are higher rates of depression (Apinuntavech et al. 2012; Blinn-Pike et al. 2010; Hanss et al. 2015), increased risk of suicide attempts (Blinn-Pike et al. 2010) at least for female gamblers (Feigelman et al. 2006). Also, associations with substance abuse; delinquency and crime; disrupted family relationships; and poorer academic outcomes are found (Blinn-Pike et al. 2010) as well as adolescent sexual behaviors (Martins et al. 2014). Chaumeton et al. (2011) found that most health-threatening behaviors were positively associated with gambling.

### Health in young people with hearing loss

Young people with hearing loss are a high-risk group when it comes to, for example, use of alcohol, tobacco and narcotics (Brunnberg et al. 2009), health-related problems (Lindén Boström and Persson 2014) and low health literacy (Barnett et al. 2011). Adolescents with hearing loss combined with another disability have heightened risk of mental health problems, school problems and substance abuse (Brunnberg et al. 2007). Students with disabilities, especially those with hearing loss are also at greater risk of experiencing low participation and exposure to bullying and threats in school than students without disabilities (Åkerström et al. 2015).

There is a lack of studies about young people with hearing loss and their gambling habits. Only one study implicitly emphasizes this (Brunnberg et al. 2007). The Örebro County region has a higher-than-average proportion of young people with hearing loss because the only national Upper Secondary School for the Deaf and Hard of Hearing in Sweden, has been located in Örebro since 1967. This explorative study therefore aims to investigate the prevalence of gambling, preferred type of gambling, and problem gambling in young people (15–18 years) with and without hearing loss.

## Method

### Data collection and participants

This study uses data from the study Life and Health—Young People, conducted in Örebro County, Sweden, in 2014. This is a total, cross-sectional survey of young people’s living conditions, lifestyles and health. All students in grade 9 (15–16 years old) of compulsory school and year 2 (17–18 years old) of upper secondary school were included in this study (n = 4888). The response rate is 79 %. For a more thorough description of the procedure (Lindén-Boström and Persson 2015).

### Measures

The gambling survey questions in this study collect data on gambling participation, preferred type of gambling and problem gambling. Gambling participation is measured with one question: Have you ever gambled? The answer alternatives are “No”, “Yes, in the past 30 days.”, “Yes, in the last 12 months” and “Yes, more than 12 months ago”. These are presented, according to earlier studies (Abbott et al. 2014), as lifetime prevalence (LTP) and present-year prevalence (PYP) (Table 1).

**Table 1:** Lifetime prevalence (LTP), present year prevalence (PYP) of gambling and problem gambling (using Lie/Bet items) categorized by with and without hearing loss, and divided into gender and age (school year) with *p* values for Chi square-tests (n = 4644)

	LTP	PYP	Bet	Lie	Problem gambling
All	25.2	19.2	3.2	2.6	4.5
Hearing loss (HL)					
None	24.4	18.9	3.1	2.3	4.3
“Mild”	35.5	24.9	4.7	6.0	7.7
“Severe”	32.4**	24.3**	0	8.1**	4.5*
With HL	35.2	24.8	4.2	6.2	7.1
Without HL	24.4**	18.9**	3.1	2.3**	4.3*
All boys^#^	37.0	29.2			
With HL	45.7	32.6	5.9	9.1	10.7
Without HL	36.2*	28.9*	5.4	3.9**	7.3
All girls^#^	13.1	9.3			
With HL	22.3	15.1	1.4	2.1	2.5
Without HL	12.5**	8.9**	0.8	0.7	1.2
All grade 9^#^	20.7	15.5			
With HL	31.4	26.3	3.2	5.7	6.7
Without HL	19.9**	14.6**	2.2	1.7**	2.9**
All year 2^#^	29.7	23.2			
With HL	38.5	23.5	5.0	6.7	7.4
Without HL	28.9**	23.2**	4.1	2.9*	5.6

* *p* < 0.05; ** *p* < 0.005, ^#^ *p* < 0.001 between boys and girls and grade 9 and year 2

Questions on preferred gambling forms were asked: Which types of gambling have you participated in during the last 12 months? The answers could be Internet gambling, slots, lottery, betting on sports, poker/cards with friends, or other forms of gambling. Questions on gambling frequency follows gambling forms, the five answers provided for selection are: “several times a week”, “several times a month”, “once a month or more seldom”, “have not done in the past year” and “have never done”.

Using the Lie/Bet Questionnaire (Johnson et al. 1997) problem gambling is measured with two items: “Have you ever felt the need to bet more and more money?” and “Have you ever lied to people who are important to you about how much you gambled?” with the answers “yes” or “no”. Problem gambling is defined as answering yes to one or both questions, in accordance with earlier studies using these items (Algren et al. 2015; Ekholm et al. 2014; Johnson et al. 1997; Götestam et al. 2004; Hansen and Rossow 2008).

The question on hearing loss was: Do you have any of the following impairments: “Hearing loss” with the answers “yes, mild”, “yes, severe” and “no”.

Questions on sex and age (grade in school) were used to indicate socio-demographic status. Other questions are “Where were you/your parents born?”. Parents’ employment was determined with the question “What do your mother/father do?”. The adolescents’ living situation was determined with the question “Whom do you live with?” and the answer were dichotomized into “Live with at least one parent” and “Live apart from parents”. The school achievement was measured with one question “Have you failed any subjects?” and was dichotomized into “Not failing any subject” and “Failing at least one subject”.

### Statistical analysis

In this study descriptive statistics (frequencies, percentages and chi square tests) were used. Multiple logistic regression was used to find the predictors of gambling and problem gambling. All statistical analyses were performed using statistical package IBM SPSS Statistics 22.

## Results

### Gambling participation

One-fourth (25 %) of all students have gambled during their lifetime and one-fifth (19 %) during the present year (Table 1). Boys have gambled to a greater extent than girls (37 % vs 13 %), and older students’ more than younger students (30 % vs 21 %). More students with hearing loss gambled during their lifetime (35 %) and in the present year (25 %) than their hearing counterparts (lifetime: 24 %; present-year: 19 %) both among boys and girls, and grade 9 and year 2 students.

### Preferred types of gambling

The most common type of gambling activity among all adolescents is buying lottery tickets (60 %). The second most common gambling form is playing poker or card games with friends (more than 40 %). More young people with hearing loss than without hearing loss was playing poker (56 % compared to 40 %, *p* = 0.004). Gambling on the Internet was relatively least common among all adolescents (less than 30 %).

### Problem gambling

Using the Lie/Bet Questionnaire (Johnson et al. 1997) 7.7 % of the students with hearing loss compared to 4.5 % (*p* < 0.05) of the normal hearing students were categorized as problem gamblers (Table 1).

Logistic regression analyses showed that significant predictors of gambling were age, (OR = 1.4, 95 % CI = 1.2–1.6), hearing loss (OR = 1.5, 95 % CI = 1.2–2.0), gender (OR = 0.3, 95 % CI = 0.2–0.3), that the students did not know if their parents were employed (OR = 1.7, 95 % CI = 1.0–2.8) and that they were living apart from their parents (OR = 1.5, 95 % CI = 1.0–2.1) (Table 2). Significant predictors for problem gambling were age (OR = 1.7, 95 % CI = 1.2–2.3), school grades (OR = 2.0, 95 % CI = 1.5–2.7), gender (OR = 0.2, 95 % CI = 0.1–0.2) and living away from their parents (OR = 1.9, 95 % CI = 1.1–3.4).

**Table 2:** Multiple logistic regression showing odds ratios (with 95 % CI) of having gambled and being identified as problem gambler adjusted for age, gender, hearing loss and socio-demographic variables

	n	Gambling		Problem gambling	
		OR	95 % CI	OR	95 % CI
Age					
Grade 9	2144	1.0		1.0	
Year 2	2185	1.4**	(1.2–1.6)	1.7**	(1.2–2.3)
Gender					
Male	2182	1.0		1.0	
Female	2147	0.3**	(0.2–0.3)	0.2**	(0.1–0.2)
Hearing loss					
Without HL	4014	1.0		1.0	
With HL	315	1.5**	(1.2–2.0)	1.5	(0.9–2.3)
Parents’ employment					
Yes, both work	3256	1.0		1.0	
One works	855	1.1	(0.9–1.3)	1.3	(0.9–1.8)
Neither works	144	0.7	(0.5–1.1)	1.3	(0.6–2.8)
Don’t know	74	1.7*	(1.0–2.8)	2.1	(0.9–4.7)
Child and parents’ birth country					
All born in Sweden	3145	1.0		1.0	
Child born in Sweden and at least one parent born outside Sweden	745	0.9	(0.7–1.1)	1.3	(0.9–2.0)
All born outside Sweden	439	0.8	(0.6–1.1)	1.5	(0.9–2.3)
Living situation					
Live with at least one parent	4179	1.0		1.0	
Live away from parents	150	1.5*	(1.0–2.1)	1.9*	(1.1–3.4)
Grades					
Not failing any subjects	3157	1.0		1.0	
Failing at least one subject	1046	1.1	(0.95–1.3)	2.0**	(1.5–2.7)

* *p* < 0.05; ** *p* < 0.005

## Discussion

### Main findings

There is a paucity of research in this under-examined area. This is the first study explicitly focusing on gambling in young people with hearing loss. Hence, this study was an exploratory study with results to be further verified in future research.

This study helps increasing our understanding of gambling behavior among adolescents with hearing loss. The students with hearing loss have higher prevalence of lifetime and present year gambling than students without hearing loss. As for young people without hearing loss, boys gamble more than girls. More young people with hearing loss than without hearing loss was playing poker with friends. More students with hearing loss compared to normal hearing students were defined as problem gamblers. Hearing loss was a significant predictor for gambling participation, but not for problem gambling when adjusted for other predictors.

### Result discussion

For the entire sample, around 25 % of the young people in this study have gambled during their lifetime. This is in line with a recent study in Sweden (Svensson 2014). Slightly below 5 % were found to be problem gamblers, which also is in line with earlier studies (Abbott et al. 2014; Blinn-Pike et al. 2007; Olason et al. 2011). This study provides evidence-based data on gambling among young people with hearing loss. Only one similar study was found (Brunnberg et al. 2007). Around 8 % of the young people have hearing loss in this sample. More of this group have gambled during their life-time. They also had a higher rate of past-year and lifetime problem gambling than young people without hearing loss. So why do young people with hearing loss gamble more than young people without hearing loss?

A protective factor that has been emphasized in problem gambling is parental monitoring (Hanss et al. 2015). More young students with hearing loss lived apart from their parents to attend the national upper secondary school for the deaf and hard of hearing. They lacked parental monitoring and support. When adjusted for living apart from parents, young people with hearing loss still have a higher LTP for gambling. Problem gambling are not explained by the hearing status of the adolescents, but by their living situation. In this study, living apart without monitoring and support from one’s parents seems to be a risk factor for problem gambling.

In this study, failing one or more school subjects appears to predict problem gambling among students with and without hearing loss. It is also important that prevention starts at an early age (Fröberg et al. 2015).

Previous research shows that the increased accessibility of Internet and hence the increased availability of gambling opportunities (Volberg et al. 2010) would potentially make Internet gambling one of the most common types of gambling among young people, especially among the group with hearing loss. However, in this study Internet gambling was the least preferred gambling activity. More young people with hearing loss were fond of playing cards together with their friends than the normal-hearing youth.

### Methodological discussion

In this study the Lie/Bet questionnaire (Johnson et al. 1997) has been used instead of the more extensive Problem Gambling Severity Index (PGSI) questions. The reason for this is that the gambling questions in this study were part of a larger health-related survey, and could therefore not be that extensive. However, that is a common and cost effective way to reach many students in one survey.

A strength of this study, as far as hearing loss youth are concerned, is the success to recruit a considerable proportion of young people with hearing loss (n = 318). The study also has a quite high response rate, and the target research population included Swedish students recruited in both urban and rural municipalities.

This study did not include a measure of how much money the young people wagered, which could be included in future research. Gambling with a small amount of money could involve a different level of risk than gambling with a large amount of money among young students. More questions are needed to examine young people’s gambling habits and the contexts where they gamble and qualitative research methods may also be considered to achieve triangulation.

### Conclusions

Many studies report that problem gambling is not isolated from other lifestyle behaviors (Chaumeton et al. 2011; Dickson et al. 2008; Swedish Public Health Agency 2014). A comprehensive preventive approach invloving parents and schools could be one way of working. To conclude, this exploratory study has made contribution to expanding our understanding of the relationship between gambling problems and hearing loss but more research is required. Knowledge about health and lifestyle behaviors in minority groups, such as adolescents with hearing loss, is a prerequisite to increasing equality in health regardless of impairments.
